# Talk to us! Communication is a key factor in improving the comfort of MRI research participants

**DOI:** 10.1111/hex.13217

**Published:** 2021-05-05

**Authors:** Rebecca S. Dewey, Claire Ward, Andrea Junor, Adele Horobin

**Affiliations:** ^1^ Sir Peter Mansfield Imaging Centre School of Physics and Astronomy University of Nottingham Nottingham UK; ^2^ National Institute for Health Research (NIHR) Nottingham Biomedical Research Centre Nottingham UK; ^3^ Hearing Sciences Division of Clinical Neuroscience School of Medicine University of Nottingham Nottingham UK; ^4^ Patient co‐facilitator Nottingham UK; ^5^ Radiological and Imaging Sciences Division of Clinical Neuroscience School of Medicine University of Nottingham Nottingham UK; ^6^ Nottingham University Hospitals NHS Trust Queens Medical Centre Derby Road UK

**Keywords:** communication, community involvement, magnetic resonance imaging, patient comfort, patient involvement

## Abstract

**Context:**

Magnetic resonance imaging (MRI) is an invaluable diagnostic and research tool. Having an MRI scan is not always comfortable and may deter people from taking part in MRI research. Maximizing comfort during scanning will improve participants’ experiences and image quality.

**Objective:**

To define which factors improve comfort during an MRI scan for research by asking people who have participated in MRI research.

**Setting and participants:**

People who had participated in MRI research during the past two years were invited, as ‘public advisors’ to discuss their experiences together and agree on which factors are most important in ensuring comfort while participating in MRI research.

**Results:**

Public advisors ranked researcher‐participant communication as the most important factor. In response, an example script to guide MRI researchers in communicating with participants was developed through close consultation between research staff, public advisors and the public. This outlines the often‐missing information necessary to convey to participants, including explaining the reasons behind instructions, managing expectations, providing reassurance, encouragement and progress updates during scanning.

**Conclusions:**

Drawing upon personal experiences as MRI research participants, public advisors highlighted the importance of effective and on‐going researcher communication throughout. The example script may be used as a training tool for researchers to help ensure participants’ comfort during scanning.

**Patient and public contribution:**

All contributors had previously taken part in MRI research. The project was co‐designed, co‐delivered and co‐authored with a public research partner. Public advisors agreed key factors of importance. External public reviewers and public advisors reviewed example script drafts.

## 1 INTRODUCTION

The NHS Long Term Plan aims to increase the number of people registering to participate in health research in England to one million by 2023/24.^1^ The National Institute for Health Research reported that over 870,000 people took part in health and social care research across England in 2018/19, an increase of over 140,000 from the previous year.^2^ However, timely recruitment and retention of research participants is a significant challenge across health and social care research. Just 56% of publicly funded health technology assessment (HTA) randomized controlled trials active between 2004 and 2016 achieved final recruitment targets, with an average retention rate of 89%.^3^ There was considerable variation in the consent, recruitment and retention rates between trials and very limited evidence of recruitment success improving over the years reviewed.^3^ Uncertainty around how best to recruit and retain participants is further evidenced by Clinical Trials Unit Directors who listed recruitment and retention as amongst the top 3 priorities for research in trial methodology.^4^

The National Institute for Health Research states the importance of delivering a positive participant experience to ensure that more people take part in research, and that those who do take part will do so again. To inform progress, the NIHR conducts an annual survey of recent research participants to gather feedback on their experience. In the 2018‐19 survey, over 90% of respondents agreed they had a positive experience of participation.^5^ This is encouraging, but the reports state that there remain opportunities to improve the participant experience through working with patients and the public to understand local factors and plan improvements together. Such partnerships are commonly referred to in the UK as patient and public involvement (PPI). PPI is defined as an active partnership between the public and researchers in the research process, rather than the recruitment of people as ‘participants’ in research. PPI is research carried out *with* or *by* members of the public rather than *to*, *about* or *for* them.^6^ PPI can benefit many aspects of research through improving its relevance and appropriateness to patient needs.^7^ A recent review concluded that PPI can improve participant recruitment to clinical trials.^8^ While there is insufficient evidence for the impact of PPI on participant retention,^8^ the PRioRiTy II (Prioritising Retention in Randomised Trials) James Lind Alliance Priority Setting Partnership shortlisted ‘How does involvement of patients/the public in planning and running trials improve retention?’.^9^ There is clearly an interest in learning more about the role that PPI can play in overcoming the on‐going challenges of recruitment and retention in clinical trials.

PPI may have a valuable role in shaping practice around the needs of participants who take part in magnetic resonance imaging (MRI) research. MRI is the preferred, gold‐standard diagnostic imaging technique for addressing numerous clinical questions. MRI is highly versatile and non‐invasive, and as such provides many valuable research tools. Many groups worldwide perform research involving MRI, addressing research questions ranging from improving the methods used to generate images, through to assessing the causes and progress of disease and the effects of treatments. There is no standard protocol for interaction with research participants in MRI studies, and experiences are likely to vary by site and by research group.

Preparing for and having an MRI scan is known to be a source of anxiety.^10^ The process may involve experiencing long scan times, loud sounds and vibrations emanating from the scanner, sitting or lying in an uncomfortable position or enclosed space, staying still for long periods or being asked to remove jewellery and clothing containing metal. Participants also experience a level of sensory and social deprivation during imaging. This has been reported anecdotally and in surveys.^11^ These factors can all contribute to an uncomfortable experience. Further, if a participant is not relaxed, it can be more difficult for them to stay still during scanning, adversely affecting the quality of any images acquired. Recent work has shown that cancer patients invited to take part in MRI research consider the number and duration of scans when deciding whether to consent. They are greatly influenced by the information provided by the researcher when making their decision, as well as their own personal circumstances.^12^

Working with members of the public who have had recent experience of taking part in MRI studies, we sought to fully characterize barriers to comfort, both physical and psychological, when undergoing an MRI scan for research. This was with a view to identifying how best to make taking part as comfortable as possible. We aimed to produce a set of guidelines for optimal participant comfort, together with advice about how to implement these guidelines. Initially, these outputs were to be applied within the University of Nottingham's Sir Peter Mansfield Imaging Centre, with the intention of encouraging uptake more widely after trial use and further feedback. We also aimed to explore and illustrate how involving patients and the public can improve the participant experience, specifically within the context of imaging research. This will act as a useful guide for others in how involving patients and the public can improve research and tackle the on‐going issues around recruitment and retention.

## 2 METHODS

The scope of the project was to fully characterize what patients and the public, who undergo MRI as research participants, perceive as barriers to comfort during the MRI scanning process. This was based on the methodology of involving patients and the public as research team partners as defined by INVOLVE.^6^ We also consciously involved members of the public at different, and complementary, levels of involvement throughout the project, in the roles of project team member and co‐facilitator, public advisor and external public reviewer, outlined below. The MRI scanning process is defined as the period spent in the MRI department on the day of the scan. This article was written in accordance with the guidance for reporting involvement of patients and the public (GRIPP2) checklist.^13^

### 2.1 Patient and public involvement

#### 2.1.1 Roles

This project involved patients and members of the public in different roles, as outlined below:

##### Project team member and co‐facilitator

As someone highly skilled in including the patient and participant perspective in research and with lived experience of undergoing MRI for diagnostic and research purposes, CW was invited to join the project team at the earliest opportunity. This is so that the project could be devised and co‐produced with someone who has relevant lived experience. CW had previously worked with the research group in co‐producing the East Midlands lay assessor training programme with AH previously.^14^ RD, AH, AJ and CW formed the project team.

##### Public advisors

The project team sought a group of public advisors numbering between 6 and 8 to keep the group size manageable so that everyone could have a say. Care was taken to minimize the burden of travel to participants. Consequently, the project team recruited six members of the public local to the Sir Peter Mansfield Imaging Centre in Nottingham, with lived experience of taking part in MRI research in the past two years. These participants joined the project as public advisors. We aimed to achieve a broad mixture of lived experiences relating to MRI research in the room, including different MRI scanners, research questions and body areas. Advisors’ MRI experiences included university departments and national testing centres, and undergoing scans of the head, torso and abdomen as patients with pathology and as healthy control participants. Advisors were aged 18 years or over. Prior PPI experience ranged from highly experienced in PPI to being completely new to PPI. Public advisors played a pivotal role in identifying the barriers to comfort, and how to overcome these barriers.

##### External public reviewers

The project team also invited a separate group of members of the public to offer an independent review of material produced during the project. This group, who had individually experienced MRI as either research participants or patients, had attended an unrelated workshop in November 2019, organized by the NIHR East Midlands Clinical Research Network, with the broader remit of gathering views on what aspects of imaging research are important to participants.

#### 2.1.2 Activities

##### Planning

The project team agreed that bringing public advisors together to encourage sharing of experiences and perspectives would be the best way to inform the development of guidelines for optimal patient/participant comfort. A half‐day discussion meeting was planned to gather information and to explore and agree the next steps with our public advisors. We agreed that:

- The project team had the capacity and could host a meeting with between 6 and 8 public advisors.
- Public advisors would be organized into small, round table groups to discuss the issues.
- Each small group would have their own facilitator, in the form of a project team member.
- CW would play an equal role in co‐facilitating the meeting, and, together with AH, introduce the concept of PPI and the purpose of the meeting.
- The meeting would have the objective of answering well‐defined questions, so it is clear to the advisors how they could contribute.
- Advisors would be asked if, and how, they would like to contribute feedback and participate in any next steps.
- The project team would seek opportunities to continue the advisors’ involvement, beyond the first meeting.
- The meeting would be held at the Sir Peter Mansfield Imaging Centre, affording the opportunity to offer the advisors a tour of the facilities.

##### Meeting with public advisors

**FIGURE 1:**
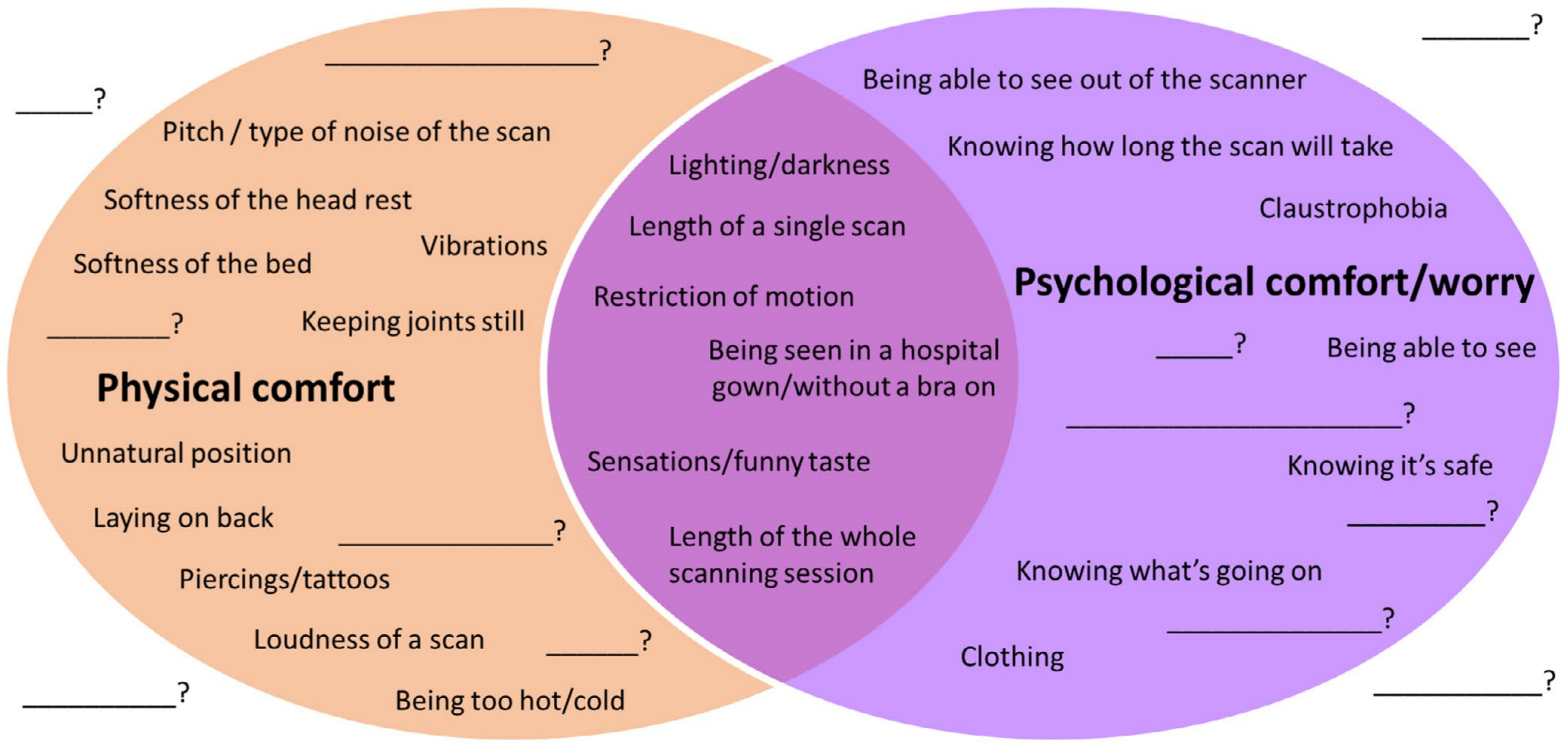
Slide from the overview of proposed aims and objectives, presented to the public advisors at the start of the half‐day meeting, with the purpose of forming a starting point for discussion. The items listed were identified by the facilitators as being commonly anecdotally associated with inconvenience or discomfort while undergoing MRI or in proximity to an MRI scanner

The project team co‐facilitated the meeting. Six public advisors attended, who had been recruited through the process described above. The advisors were split into two groups of three, with each group being allocated two facilitators: a chair and a note taker. Two additional members of the public attended in the capacity of carers for a recruited advisor. Although unplanned, carers were also welcomed into the debate, to enrich the perspectives and maintain a sense of inclusion and openness. Attendees (advisors and carers) were provided with a basic introduction to PPI by AH and CW. RD presented an overview of the proposed aims and objectives, including a brief introduction to the current understanding of factors reported to impact comfort during MRI scanning. This included visual prompts such as the informal word cloud of factors that are thought to influence comfort during MRI, shown in Figure 1, to kick‐start discussions. Within their groups, attendees were asked to give their initial thoughts and perspectives on being a participant in MRI research. Facilitators were available to chair and take comprehensive notes. After attendees shared their experiences, each group was asked to summarize an agreed set of factors or themes. Once these factors/themes had been agreed upon, attendees were asked to rank them in order of importance. At each stage, facilitators took comprehensive handwritten notes that were visible to the attendees throughout so that any inconsistencies or errors could be immediately corrected. Finally, attendees were asked to complete a short debrief form indicating if they wished to be contacted by the project team in the future.

##### On‐going feedback and continued involvement

At the end of the meeting, attendees were invited to leave written feedback of their experience by completing a short form asking them to highlight two things that they had enjoyed or valued during the meeting, and one thing that could have been improved. A longer feedback form was sent to participants a few days after the meeting. This included positive feedback from the project team on how useful input from the advisor group had been, the main conclusions drawn and an outline of the next steps. The feedback form provided a further opportunity for the public advisors to offer more detailed feedback on the meeting outcomes and to comment on the conclusions. The public advisors were also invited to contribute feedback via email on material produced subsequently.

### 2.2 Developing guidance on optimizing comfort for MRI research participants

Factors prioritized by public advisors, and any further feedback they gave after the meeting was compiled and shared amongst the project team. Following this, RD drafted a script outlining the information that participants had reported being missing in their experience of participating in MRI research. The target purpose of the script was to form a training or reference aid for members of research staff undertaking MRI of patients and the public, to remind them of key information that needs to be conveyed. On the basis of discussion that arose during the half‐day meeting, the script started from the point of recruitment into the study, describing the process of MRI, the measures taken to ensure participant safety and image quality, and, crucially, the reasoning behind them. The script continued to outline information necessary to be conveyed during the research participation appointment, again, crucially, at each point explaining what the researcher is doing and why. The script emphasized the necessity of continuous communication with the participant, describing what was about to happen and how long it would take, and providing reassurance. Finally, the script outlined a debrief to take place following the completion of imaging, prior to the participant leaving the department. RD consulted the project team and colleagues experienced in conducting MRI scans on patients and the public in the formation of this script and the subsequent editing process. The script was then shared with the public advisors and external public reviewers for their review and written feedback.

### 2.3 Example script feedback

Feedback was requested on the following:

1. Are there any parts of the script that you think participants may find upsetting or distressing?
2. Are there any parts of the script that you think participants may find inappropriate?
3. Are there any parts of the script that you feel are unclear or ambiguous?
4. Are there any parts of the script that you feel are unnecessary?
5. Do you think there is too much information in the script?
6. Can you think of any information that should be in the script that we do not include?

Amendments were made to the script following this feedback.

## 3 RESULTS

### 3.1 Meeting with public advisors

At the half‐day meeting, the two groups of attendees were asked to rank factors in order of importance. Facilitators took comprehensive handwritten notes during the discussion, and attendees were able to read and make additions and corrections to these notes throughout to ensure their accuracy. However, facilitators were careful not to steer the conversation in any direction. As such, one group produced a list of six ranked themes for factors of importance to comfort when participating in MRI research, while the other group produced a list of seven factors of equal importance (ie no ranking order). These two sets of factors are given in Table 1. The six factors in order of importance were communication, claustrophobia, hardness of the bed, length of scan, positioning of body and the temperature of surrounding areas. The seven factors of equal importance were the provision of prior information, communication during the scan, the audio quality of the communication system, being told how best to communicate with the researcher during scanning, the suitability of clothing worn during scanning, the temperature of the general environment and time pressures.

**TABLE 1:** Factors of importance to comfort when participating in research involving MRI

1. Communication—pre‐information, managing expectations, reassurance before and during scan 2. Claustrophobia 3. Comfort of bed—too hard 4. Length of scan—mitigate effects of long scan, breaks, countdown timer visible 5. Positioning of body—for example relieve spinal tension by pillow under the knees 6. Temperature—of surrounding areas, not necessarily of scanner itself	= Prior information—opportunity to ask for questions, elaboration, clarification = Communication during scan—encouragement, updates on duration, progress = Clearer audio for communication—noise‐cancelling headphones, even visual communication = Knowing how to communicate and confidence that you can be heard during scan = Clothing to avoid embarrassment particularly if needing toilet facilities between tests = Temperature of general environment = Time pressure—rushed into it, no time to adjust, ask questions

Note

Lists on the left and right reflect the responses of the two groups of three participants each. Factors/themes produced by the group on the left are ranked in order of importance, whereas factors/themes produced by the group on the right are of equal weighting and are not ranked in any order.

The need for effective communication and the temperature of surrounding areas were the only factors given importance by both groups. Contrary to our expectations that claustrophobia, physical comfort and time‐related issues, such as the number of scans and scan duration, would be the main discussion points, it was unexpected that both groups would speak at length about communication issues, both pre‐scan and during the scan itself. In short, our advisors wanted to know what to expect beforehand and what was happening during the scanning procedures. Their experiences showed that they were not always given this information when they would have found it helpful.

Conversely, when this information is provided, participants report having a positive experience, as illustrated by one of the Patient Advisors, John Wood: ‘*The MRI procedure was simple, and having been advised of the likely noises it proceeded in a relaxed manner. On one of my previous scans I was invited to bring along a personal music CD which was played to me during the scan; this was a very thoughtful idea. This aided the relaxation and provided confidence that the operators were concerned for my wellbeing as well as ensuring that the scan proceeded without problems’*.

As shown in Table 1, one of the groups prioritized communication as the most important issue affecting comfort. For the other group, 4 out of the 7 issues shortlisted referred to aspects of communication. It was this emphasis and focus on communication by our public advisors that directed us to produce guidance for imaging researchers to improve their procedures for communicating with participants, both before and during participation in the study.

### 3.2 On‐going feedback and continued patient and public involvement

Feedback given by public advisors at the meeting was broadly positive. They enjoyed the experience, learning more about MRI and others’ experiences of it and welcomed the opportunity to contribute to research. They expressed the hope that their experiences would help improve future participants’ experiences, indicating a distinctly altruistic motivation to involvement. Logistically, they reported that organization and communication leading up to the meeting was good. They also welcomed the frequent breaks during the meeting. Difficulties in parking at the venue were reported, as unfortunately, we were not allowed to reserve spaces for participants. In addition, some would have valued more information and ‘scene setting’ at the beginning of the meeting. It was also noted that it would be useful to increase the numbers of people involved and to widen the ethnic, and possibly religious diversity of those present, as five out of the six public advisors were white. Some public advisors reported difficulties in listening to group discussions, over conversations from the adjacent group, indicating that using separate breakout rooms may be better for future events.

Written feedback provided by the project team on the advisors’ input at the meeting outlined the need to consult patients and members of the public, and how the work undertaken would not have been possible without the help of the public advisors. It was acknowledged that there was a strong agreement across the group, and the main conclusions were outlined. We informed that those who had said they were willing to be contacted would be asked to help further down the line with the development of the materials that resulted from the research.

### 3.3 Developing guidance on maximizing comfort for MRI research participants

All six public advisors were sent a draft of the example script, and a copy of the questions listed in the Methods section, Example Script Feedback. Additionally, 30 external public reviewers (as described in the Roles section, above) were also sent the script and the questions. These two groups of people were initially sent electronic copies of the materials in pdf and were told that they could request paper copies if they preferred. Written responses (electronic or in paper format) were received from a total of six individuals, comprising four of the public advisors and two external public reviewers. Responses were anonymized immediately upon receipt, and all taken at equal weighting.

The script was amended to incorporate all suggested edits where these did not directly contradict the suggestion of another respondent. Some feedback was conflicting, with suggestions to either shorten the script by removing some information out or to add more information in. The majority view, however, was in favour of adding more detailed information. Many suggestions comprised amending the document for clarity. This included adding additional lines of explanatory information such as ‘MRI stands for magnetic resonance imaging and is a way of taking pictures of the inside of your body’, and ‘if you move during scanning, it will affect the quality of the images’. Feedback also highlighted jargon and included suggestions for plain English alternatives. For example, replacing ‘scrubs’ with ‘gown’ or ‘clothing’ and clarifying ‘removable metal’ using the examples ‘jewellery and piercings’. It was suggested to add ‘if applicable’ to statements that may not apply to all participants, such as the removal of a bra before scanning. Other feedback included covering themes that had been overlooked in earlier drafts of the script, and that were highlighted by respondents, such as offering the participant a final opportunity to use the toilet before scanning commences, explaining that the scanner bed can be removed from the room in case of an emergency and explaining any features of the intercom system that limit when the participant's voice can be heard by the researcher. The full, final script is available in Supplementary Material S1.

## 4 DISCUSSION

This work aimed to involve members of the public with lived experience of being participants in MRI research to lead us in identifying what affects comfort during MRI scanning for research purposes. We intended to achieve this by asking patients and the public with lived experience of being participants in MRI research how their comfort could have been improved while they were taking part in MRI research. We intended that the impact of this work should expand, sequentially. This would comprise applying the developed script as a tool to improve communication in research involving human participants carried out in the local department, with the intention of encouraging uptake more widely after a trial period use and further feedback.

The initial step of the information gathering meeting was intended only as that the mechanism of finding a direction. The project team had hypothesized that the public advisors would raise issues relating to physical comfort, such as a trade‐off between the length of individual scans and the entire scanning duration, or the noise levels produced by the scanner during certain scans. Instead, the process of information gathering highlighted a significant shortfall in practice and procedure, in that advisors emphasized the need for receiving clear, detailed and continuous communication from the researcher throughout participation in the study.

Our project team member and patient co‐facilitator, CW, illustrated her own motivation for conducting this research: ‘*As a patient who sustained a serious lifelong injury 27 years ago I have had repeated MRIs on my spine, and also my chest, head and shoulders. These have been undertaken at a few different sites. Over the years I have seen changes in both the process and procedure of undergoing MRI, and areas such as providing pillows and making sure people are comfortable have improved somewhat, however there is still room for improvement where communication is concerned. I was very pleased to be asked to co‐facilitate this meeting with members of the public and was also pleased when the group placed such importance on communication as the overriding issue. Both clinical and research staff can do something about this and improve communication throughout the entire MRI process, and we have been able to make valid suggestions for them to take on board’*.

As such, the work changed in direction, to one of improving the communication shortfall by producing a standard operating procedure for communication with research participants undergoing MRI in the form of a script, or list of advised minimum information requirements for interactions with participants. This was directly fed into by members of the public and patients who had participated in MRI research. This was also in agreement with previous research demonstrating the importance of the quality of study materials in a potential participant's decision to take part in a study and that better quality materials are likely to improve patient tolerance and acceptability.^12^

The overwhelming feedback from public advisors and from external public reviewers was that the work was highly needed and relevant and that the script, if followed, would make a valuable improvement to the psychological comfort and peace of mind of those participating in MRI research. The majority of feedback also strove to add more information into the script, as, on reflection of their own first‐hand experience of participating in MRI research, they would have benefitted from more information rather than less. This was not unanimous however, and some feedback (very much in the minority) was that there was an excess of information. This feedback was also taken on board, in that the script is broken down into subsections, with only the information relevant to that subsection included. Throughout, the language has been made clear, universal and unambiguous.

The intended scope of this work was that it should improve the comfort of patients and the public, in their participation in research involving MRI. As such, the focus has been kept solely on MRI performed for the purposes of research, and not for clinical investigative purposes. This is a limitation in the scope of the work that served to reduce the size of the question, but also reduces the applicability of the results. The pressures of a clinical department are very different to those of a research department. Clinical departments have much higher patient throughputs and much shorter appointments. Further work will need to be done to improve the comfort of patients undergoing MRI in a clinical setting, where additional anxieties around patients’ health or the outcome of their diagnosis may need to be acknowledged and addressed. Measures needed to improve the participant experience in research involving other imaging modalities will require further work also, as many of the inconveniences associated with other modalities were not discussed in the present context.

Further, the work presented here is limited in that the research tool developed in the form of the example script only addresses distress caused by inadequate communication on the part of the researcher conducting the study. Focusing on a specific and highly prioritized element of comfort kept the project manageable and produced a practical tool to help improve future participant experiences. However, the present article does not attempt to address the remaining aspects of either psychological (for example claustrophobia) or physical (hardness of scanner bed, duration of scan, unnatural positioning of body, temperature) discomfort reported by public advisors in the initial consultation. Many recent advancements have worked towards speeding up image acquisition.^15^, ^16^, ^17^ Many hardware improvements over the years have gradually worked to reduce the confinement, physical discomfort and claustrophobia of MRI due to increases in scanner bore width, as well as addressing acoustic noise discomfort by facilitating decreases in the loudness and duration of scans.^18^

As pointed out by one of the attendees of the half‐day meeting, there were only six public advisors involved in the first stage of the work, and there were also only six individuals who provided responses in the consultation on the draft of the example script. These small numbers reflect a further limitation of the work. A larger project, covering a wider geographical area and increased number of MRI research centres would have a wider scope and would allow for a more systematic and exhaustive approach to ascertaining what factors are important in the comfort of patients and the public taking part in MRI research. Alternatively, future work could comprise an iterative process, whereby guidance is developed, and procedures improved based on on‐going feedback.

## 5 CONCLUSION

The consultation of patients and the public who underwent MRI in the participation of research resulted in a set of factors that were considered to affect the comfort, either psychological or physical, of the participant. Many of these factors, including, where ranked, the most important factor, pertained to the degree of communication between the participant and the researcher prior to, and during, participation in the research study. We had not anticipated this outcome, highlighting the influence that patient and public involvement can play in identifying what needs to be done to improve the participant experience. Whether this can translate into improving participant retention in clinical trials is a question certainly worth investigating, as highlighted by the PRioRiTy II (Prioritising Retention in Randomised Trials) James Lind Alliance Priority Setting Partnership.^9^

Further to this, an example script was developed to assist researchers in the effective communication of many further aspects of the participant's physical and psychological comfort during scanning. This script underwent further editing in consultation with patients and the public. This script is available (see Supplementary Material S1), for use as an educational tool to assist in the training of researchers conducting MRI involving patients and the public.

## CONFLICT OF INTEREST

This research was funded by the NIHR Nottingham Biomedical Research Centre. The views expressed are those of the authors and not necessarily those of the NHS, the NIHR or the Department of Health and Social Care. No conflicts of interest are declared.

## Supporting information

Supplementary Material

## Data Availability

No further data were generated in this patient and public consultation.
