# Supplementary material for: Talk to us! Communication is a key factor in improving the comfort of MRI research participants
Source: Health Expect. 2021 May 5;24(4):1137–44. doi: 10.1111/hex.13217 (PMC8369077; doi:10.1111/hex.13217)
Supplement: Supplementary file 1 — Supplementary Material [file HEX-24-1137-s001.docx]

**Example Script Draft**

**E-MAIL / PHONE CONVERSATION BEFORE DAY OF APPOINTMENT:**

**Introduction and background**

- Thank you for your interest in the study
- We’re conducting research into <explain area of research>

**What the participant will be asked to do**

- In order to do this, we are asking people to have an MRI scan
- MRI stands for magnetic resonance imaging and is a way of taking pictures of the inside of your body
- If you have any metal implanted in your body, you may not be able to take part in the study, so it is really important that you tell us about any surgeries or accidents you’ve had that may result in there being metal implanted in your body
- I will ask you to remove all metal from your body before going in the scanner, including jewellery and piercings
- This is because the scanner is a strong magnet and loose metal can get pulled into the scanner or might heat up during scanning
- Also, any metal in or on your body will cause distortions in the images
- <if desired, send participant the safety screening form and ask them to raise any questions at this point>
- <if applicable> I will ask you to remove your bra – if you would like to wear/bring a bra or top that doesn’t have any metal (including any clips), you are welcome to do so
- Sometimes there is metal hidden in clothing, such as in antibacterial silver sportswear – please don’t wear this for your appointment
- <if necessary> There is metal in lots of make-up, too, so if possible please don’t wear make-up, or be prepared to remove it before your scan
- I will give you some disposable clothing (scrubs) to wear
- You can wear underwear and socks underneath
- There is a secure changing room right next to the scanner
- <if needed> We will need you to be able to see a computer screen during the scan – if you would usually need glasses for this, we can provide some MRI-safe glasses, so please find out your lens prescription so we can use the correct lenses
- <if possible> If you would like to bring some music to listen to or a DVD to watch, you are welcome to do this
- Your appointment will take about <duration> hours and you will be in the scanner for about <duration> minutes of that

**Practicalities/logistics**

- <if applicable> We will pay you an inconvenience allowance of <rate per hour or per appointment>
- <if applicable> Please remember to bring all receipts with you so that we can reimburse your travel costs
- On the day of your appointment, please come to <address, post code>
- If you are coming by car, <description>
- The parking costs <give current costs>, and I recommend you get at least <number of hours>
- There are Blue Badge parking spaces <description>
- If you are coming by public transport, <description>
- If you do not use stairs, the recommended route is <description>
- If you need to contact us on the day of your appointment, please <insert contact details>

**Any questions?**

- Do you have any questions at this stage about what to wear/bring or about what we are asking you to do?
- Is there anything we can do to help? (Access requirements, improve comfort etc.)

**DAY OF APPOINTMENT, BEFORE SCANNING:**

**Check they are fit for scanning**

- Are you feeling fit and healthy today?

**Check they are comfortable with timing and practicalities**

- Your appointment will last until about <duration> – is that ok?
- Do you have enough parking/do you have to leave earlier than that for any reason?

**Confirm genuine informed consent**

- Did you read the information sheet, and do you have any questions about it?
- Do you have any questions about the consent form?
- Do you have any questions about the safety questionnaire?

**Give overview of what will happen**

- I’m going to ask you to lie in the MR scanner for about <duration> minutes
- I will attach <describe equipment> in order to measure <purpose of equipment>
- While you’re in the scanner I will ask you to keep still
- If you move during scanning, it will affect the quality of the images
- During some of the scans, I will ask you to <describe action, e.g. hold your breath>
- I will talk to you in between the scans to remind you of these instructions

**Music/DVD options**

- <if offered> did you bring any music or a DVD to entertain you during your scan?

**Give instructions on how to prepare for scanning**

- The MR scanner is a strong magnet so I will need you to get changed and remove all metal
- I’ll get you some disposable clothing – they’re in a unisex size – what size would you like?
- Here is the changing room – there is a lock on the inside of the door
- Please remove any clothing that has metal in – you can keep your underwear and socks on but please remove your bra if it has any metal in it, including the clips
- Do you have any questions about metal on your clothing?
- You can leave all your belongings in there and it will be safe
- <if participant wears glasses> You will need to remove your glasses - we will keep them safe
- <if needed> We have some MRI-safe glasses so that you can see the screen – please can you tell me your prescription?

**PREPARING FOR SCANNING:**

**Before participant lies down on the scanner bed (also before any communication aids are removed or switched off, e.g. hearing aids, cochlear implants)**

- Do you want to use the toilet before you have your scan? You won’t be able to go for the next <duration> minutes
- The experiment we are going to do is to see what happens when <purpose of experiment>
- I’ll ask you to lie in the MR scanner for <duration> minutes total
- I will ask you to keep as still as you can during scanning
- There will be <number> scans in total, lasting <durations> each
- I will talk to you between scans to tell you what’s coming up and let you know how you’re getting on
- <if possible> If you need to move between scans, i.e. when the scanner is not scanning and I am talking to you, you can do this, but please let me know as I might need to make some adjustments
- I won’t be able to hear you while the scanner is scanning
- I will give you an alarm buzzer to hold – if there is anything wrong or you feel unwell or uncomfortable, you must squeeze the buzzer to alert me, then I can come and fix the problem, or get you out of the scanner
- If we need to, we can get this whole bed out of the room with you on it, so there’s no need to feel that you’re trapped inside
- We’ll get you comfortable beforehand – please help me to do this by telling me how to make you comfortable and telling me anything I can move or give you to make you more comfortable – remember you will be lying in the scanner for <duration> minutes and I want you to be able to keep as still as you possibly can so it’s very important that you are comfortable
- During scanning, the scanner makes loud vibrating noises as the magnetic field changes
- Therefore, I will give you some earplugs to put in in order to protect your hearing
- You need to squeeze the earplugs into a very thin cylinder and put them in your ear canal – you may need to hold them in place while they expand
- <watch participant to check they have done this correctly, or ask if they have used them before, demonstrating on yourself or helping the participant if needed>
- <raise voice>
- Do they feel like they’re blocking out the sound effectively?
- I will also give you some ear defenders to put on
- Please lie down on the scanner bed

**Make the participant comfortable**

- Would you like a foam wedge under your knees to keep your hips at a slight angle?
- <after adding each item of equipment> Is that comfortable? Would you like me to move it to make it more comfortable?
- <if using respiratory bellows> it should feel tight but should not restrict your breathing – does it feel ok? Please take a deep breath to make sure
- Do you have enough padding around/under your head?
- Would you like an extra pillow/layer of padding in the coil?
- Would you like a blanket over your arms or legs?
- Are you warm enough? Do you need a blanket?
- Here is the alarm buzzer – please give it a test squeeze so you know how it works
- If there is anything wrong – you feel unwell or uncomfortable, please squeeze the buzzer to alert me

**Check equipment**

- <if using screen, position mirror for participant to see in direction of screen> once you’re in the right place you’ll be able to see the screen when you look in this mirror
- <if not using screen, position mirror for participant to see control room> you can see out of the scanner if you look in this mirror – you’ll be able to see me in the next room
- Is everything comfortable in terms of a) how you’re positioned, b) how the equipment is positioned around you?
- Are you ready to go into the scanner?
- The bed will raise upwards and go backwards
- <raise bed and move backwards to near laser point>
- Please close your eyes, I’m going to use a laser pointer to tell the scanner where I want you to be positioned
- <laser as needed>
- Thank you, you can open your eyes if you like
- Now you’re going to move backwards into the scanner
- <move bed to lasered position>

**Last comfort check**

- <shouting now>
- Are you ok? Are you still comfortable?
- <if using screen> Can you see the screen clearly? Do you want me to move the mirror/screen so that the cross is in the middle of your vision?
- I’m going to go into the next room - I’ll talk to you over the intercom in a moment

**DURING SCANNING:**

**Confirm communication channels**

- Can you hear me clearly? <adjust volume if necessary and re-check>
- I can only hear you when I’ve asked you a question, so if you need to speak to me at another time, please squeeze the button
- Are you feeling comfortable?
- Can you hear the music/see the DVD playing?
- Is it ok for me to start?
- The first scan is <duration> minutes and I will talk to you immediately after that
- I’m going to start the first scan now, please keep still
- <scan>

**During scanning (check after each scan if possible)**

- How was it?
- Thank you for keeping still
- You’re doing well
- Are you still feeling ok?
- The second scan is <duration> minutes and I will talk to you immediately after that
- For this scan I need you to <instruction, e.g. hold your breath>
- Is it ok if I start the next scan?
- I’m going to start the scan now, please keep still
- <scan>
- How was it?
- Thank you, you’re doing well
- The next scan is <duration> minutes and I will talk to you immediately after that
- Is it ok if I start the next scan?
- I’m going to start the scan now, please keep still
- <repeat as needed until all scans are completed>

**After completion of scanning**

- Thank you for completing all the scans
- I’m going to come into the room to get you out of the scanner

**AFTER THE SCAN:**

- Thank you for completing the scanning session
- How was it?
- Did you find anything strange about it?
- Do you have any questions?
- Are you feeling ok?
- You can go and get changed and put your clothes and jewellery back on
- These are your images <if any concerns about incidental findings, select a slice in which these are not visible>
- This is <anatomical feature> / this is what we’re looking for
- <if asked “is it normal?”> It is unlikely that we would notice any abnormalities since these scans are for the purposes of scientific research, and they will not be routinely looked at by a radiologist (a doctor qualified to find abnormalities in scans) – however, in the unlikely event that we do find a potential abnormality, your images will be reviewed by a qualified consultant radiologist and they would advise on how to proceed, which may include informing your GP, or referring you to a specialist for further investigation
- Would you like me to send you some pictures by e-mail?
- <if appropriate> You will receive a payment of <amount> into your bank account
- <if appropriate> This sometimes takes several weeks so please don’t panic if you don’t receive it immediately
- <check all paperwork is completed, legible and signed, before the participant leaves the building>
- Are you feeling ok? It is normal to feel a bit spaced out after an MRI scan, and we don’t want you to leave until you’re feeling well enough <if necessary, get the participant a drink and make sure there is someone to sit with them until they are ok to leave>
- If you have any concerns, please get in touch
- THANK YOU
